# Dual associations of gut and oral microbial networks with kidney transplantation

**DOI:** 10.1128/msystems.00252-25

**Published:** 2025-07-09

**Authors:** Siming Qu, Yuzheng Gu, Xiaoxue Hou, Min Wei, Mengmeng Wang, Yuya Su, Yinglei Miao, Jinlong Yang, Yang Sun, Zhong Zeng

**Affiliations:** 1 Department of Organ Transplantation Center, The First Affiliated Hospital of Kunming Medical University, Kunming, China; 2 Department of Gastroenterology, The First Affiliated Hospital of Kunming Medical University, Kunming, China; 3 Yunnan Province Clinical Research Center for Digestive Diseases, Kunming, China; 4 College of Grassland Agriculture, Northwest A&F University 12469, Yangling, China; 5 College of Agriculture, South China Agricultural University 162683, Guangzhou, China; 6 College of Life Sciences, University of the Chinese Academy of Sciences 617066 , Beijing, Beijing, China; 7 College of Forensic Sciences, Xi'an Jiaotong University 599479, Xi'An, Shaanxi, China; 8 Yunnan Geriatric Medical Center, Kunming, China; Technion, Haifa, Israel

**Keywords:** kidney transplantation, gut microbiome, oral microbiome, microbial co-occurrence networks, shotgun metagenomic sequencing

## Abstract

Gut and oral microbiomes play an essential role in the occurrence and development of kidney disease, but their changes after kidney transplantation in patients with end-stage renal disease and their relationships with host health remain unclear. Through shotgun metagenomic sequencing of fecal and saliva samples, we found that for both gut and oral microbiome, the initial loss of species diversity after kidney transplantation led to a reduction in network nodes and interactions, but strengthened the connections among the remaining species, which started to get a recovery approximately 7–14 days later. Different network modules tended to exhibit unique functions and showed different responses to transplantation. These network changes were significantly correlated with clinical indicators, especially with estimated glomerular filtration rate, suggesting that microbial networks contributed to regulating kidney function and host health from dual dimensions. Our study provides novel insights into associating microbiomes with patient recovery after kidney transplantation and offers new diagnostic strategies.

**IMPORTANCE:**

Understanding the dynamics of gut and oral microbiomes after kidney transplantation is crucial for improving post-transplant outcomes and managing potential complications. Through shotgun metagenomic sequencing of fecal and saliva samples from patients following kidney transplantation, our study emphasizes that, in addition to focusing on the various microbial species themselves, the topological properties of gut and oral microbial networks are also critically important for kidney function. We aim to explore the relationship between host health and the oral and gut microbiomes following kidney transplantation from an ecological perspective and extend to other diseases to advance the study of the microbiome and its clinical impact.

## INTRODUCTION

When chronic kidney disease progresses to end-stage renal failure, kidney transplantation becomes an important treatment option (1). Currently, the number of patients with chronic kidney disease and the number of patients waiting for transplantation worldwide are both increasing (2). Although successful kidney transplantation has improved the quality of life, morbidity, and mortality of patients compared to maintenance of hemodialysis, it still faces various challenges, such as limited access to kidney transplantation, shortage of living and decreased donors, increasing average age of donors and recipients, the presence of comorbidities in recipients, and the occurrence of post-transplant complications (3). Moreover, the currently recognized infections, injury, hormones, cytokines, growth factors, and environmental factors cannot fully explain the effects of transplant (4).

With the proposal of the gut-kidney axis, more and more studies have found that the gut microbiome is significantly influenced by kidney transplantation, and microbial changes are closely related to post-transplantation complications such as infection, rejection, diabetes, and diarrhea (5), as well as mortality (6). For example, supplementing the chronic kidney disease mouse model with *Faecalibacterium prausnitzii* can reduce kidney dysfunction, alleviate renal inflammation, and lower serum levels of various uremic toxins (7). The comparative analysis has revealed that the gut microbiome in kidney transplant recipients shows lower microbial alpha diversity (8), while the relative abundance of Proteobacteria increases (9). Meanwhile, past research has suggested that patients with organ transplantation are prone to periodontal tissue diseases, oral mucosal diseases, malignancies, and caries, which are due to the characteristic changes in oral microbiomes (10). With the decrease in kidney function, the increase in uremic toxins, urea, and creatinine in serum is related to the increase in saliva concentration, which can explain the variations in microbial structure and function in the oral cavity (11, 12). For instance, the prevalence of opportunistic pathogens such as *Enterobacteriaceae*, *Pseudomonas fluorescens*, *Acinetobacter* spp., and *Vibrio* spp. has been shown to increase in the oral microbiome of transplant patients (13). These studies suggest that in addition to the gut, the oral microbiome remains a therapeutic target after kidney transplantation. An interesting finding is that liver failure leads to an increase in overlap between oral and gut microbiomes (14). Whether this distal invasion changes over time after kidney transplantation is of interest, as this may be relevant to determining the appropriate timing of oral therapy. However, most studies to date on this topic examined the gut and oral microbiome through 16S rRNA gene sequencing approaches instead of more advanced shotgun metagenomics sequencing, which has higher classification resolution and provides valuable insights for experimental verification (15). Therefore, it is essential to explore the alterations in gut and oral microbiomes after kidney transplantation, especially at multiple time points.

In the gut and oral microecological environment, microbial interactions promote the stability and development of the ecosystem. These interactions include various forms of symbiosis, including mutualism, symbiosis, parasitism, or mutualism, all of which significantly affect the structure and function of the relevant microbial communities (16). More and more evidence shows that the cause of inflammatory diseases is related to the ecological imbalance of microbial communities (17). Constructing a microbial co-occurrence network is the preferred method to fully capture these microbial associations, thereby comprehensively characterizing the complex relationships and interdependencies among members within communities (16, 18). Past studies have shown that microbial networks can be divided into several important guilds or modules to perform specific functions and play different roles in ecosystem functions (19, 20). For instance, a recent study has revealed two competing guilds across populations and health status, which could successfully distinguish cases from controls across multiple diseases (21). Thus, using modules divided from networks can serve as a vital approach to deduce the potential relationship between microbial communities and clinical indicators. Furthermore, the links between microbial networks and organismal health are also reflected in other dimensions. A previous study has suggested that the number of edges in the gut microbial network could predict the individual responses in liver fat to exercise intervention, which breaks with the inherent idea of focusing on microbial abundance (22). Thus, it is reasonable to further explore the topological properties of microbial networks that can be useful for predicting the health conditions of patients after kidney transplantation.

This study aimed to simultaneously investigate the gut and oral microbiome through shotgun metagenomic sequencing of fecal and saliva samples, respectively, to explore microbial responses to kidney failure and transplantation. Our objectives were to assess (i) the alterations in structure and function of gut and oral microbiome, (ii) the variations in microbial networks, including network properties and module abundances, and (iii) the associations between microbial networks and clinical indicators. Taken together, we present significant novelty by linking complex gut and oral microbial ecosystems with host health.

## MATERIALS AND METHODS

### Study cohort and sample collection

**Fig 1:**
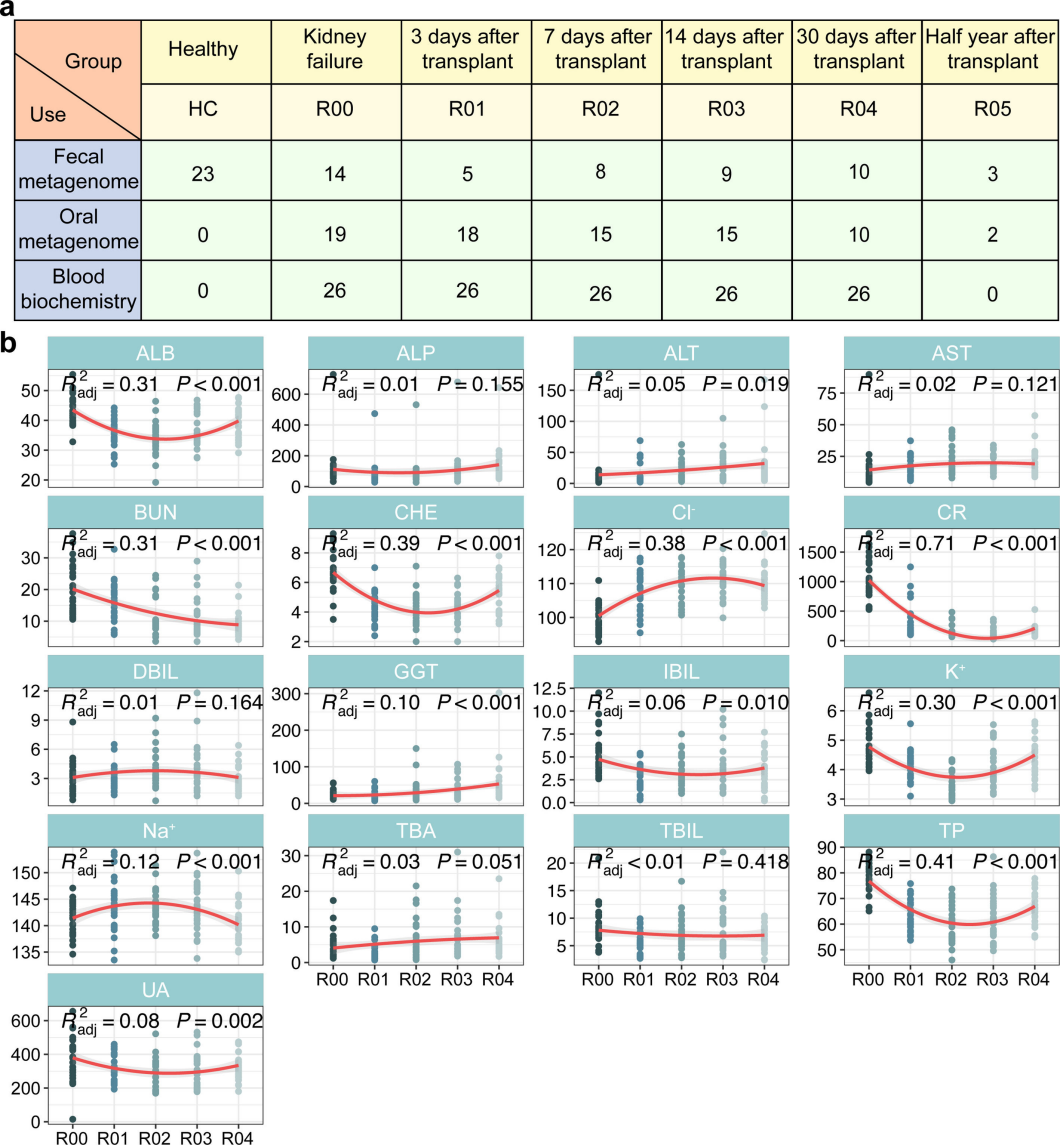
Information on gut metagenome, oral metagenome, and blood biochemistry indicators. (**a**) The sample sizes for the Healthy Control (HC), Renal Failure (R00), and post-transplant days 3 (R01), 7 (R02), 14 (R03), 30 (R04), and half a year (R05) group. (**b**) The changes in various blood biochemistry indicators, including albumin (ALB; g/L), alkaline phosphatase (ALP; IU/L), alanine aminotransferase (ALT; IU/L), aspartate aminotransferase (AST; IU/L), blood urea nitrogen (BUN; mmol/L), cholinesterase (CHE; KU/L), Cl^−^ (mmol/L), creatinine (CR; mmol/L), direct bilirubin (DBIL; μmol/L), gamma-glutamyl transferase (GGT; IU/L), indirect bilirubin (IBIL; μmol/L), K^+^ (mmol/L), Na^+^ (mmol/L), total bile acid (TBA; μmol/L), total bilirubin (TBIL; μmol/L), total protein (TP; g/L), and uric acid (UA; μmol/L). These changes are fitted by nonlinear least squares.

We collected fecal and saliva samples from adult patients with end-stage chronic kidney disease (age ≥18 years, *n* = 26) who underwent kidney transplantation at the First Affiliated Hospital of Kunming Medical University between March 2023 and March 2024. Samples were collected 1–3 days prior to transplantation and five time points after transplantation. Notably, the fecal samples were collected separately from a group of healthy individuals, without saliva samples. The number of samples under groups of healthy control (HC), kidney failure (R00), and a time gradient after kidney transplantation are shown in Fig. 1a. Specifically, fecal metagenomic samples were collected on R00, day 3 (R01), day 7 (R02), day 14 (R03), day 30 (R04), and half a year (R05) after transplantation, with 5, 8, 9, 10, and 3 samples at each time point, respectively. We also correspondingly collected 18, 15, 15, 10, and 2 oral metagenomic samples from R00 to R05, respectively (Table S1). Both saliva and fecal samples were collected in sterile containers in the hospital, and the participants ate and lived normally before collection. The samples were stored in a −80℃ refrigerator within 1 hour after collection. Likewise, samples collected from 26 recipients during R01 to R04 were used for blood biochemistry, respectively. The basic information of kidney transplant patients is found in Table S2, and detailed information on clinical indicators (blood biochemistry) of kidney transplant is shown in Table S3. The samples were also stored in a −80℃ refrigerator. In addition, all participants used antibiotics (e.g., cefoperazone-sulbactam, latamoxef, and imipenem) after kidney transplantation. We divided antibiotics into four classes, including β-Lactams, tetracyclines, sulfonamides, and oxazolidinones, and we counted the use of them by the participants at different time points. The detailed information can be found in Table S4. At the same time, all participants consistently received triple immunosuppressant therapies, including tacrolimus, mycophenolate sodium, and prednisone acetate, from R01 to R05. During this period, the dose remained essentially unchanged.

### Shotgun metagenomic sequencing

Fecal and saliva DNA were both extracted using the MagPure Stool DNA KF Kit B (Magen, China; no. MD5115-02B). DNA concentration was estimated using Qubit (Invitrogen). DNA libraries were constructed using at least 200 ng of DNA as the starting material. Shotgun metagenomic sequencing was then performed on the DNBSEQ platform to generate at least 40 million paired-end reads (100 bp in length) for each sample (23).

### Metagenomic data processing and analysis

To ensure the quality of the raw sequencing data, we utilized fastp (v0.23.2) (24) to perform a series of operations on the raw data. These operations included removing poly-G and poly-X sequences with a minimum length of 10, trimming low-quality bases from both the front and tail ends using a window size of 4, filtering out low-quality bases with a qualified quality threshold of 15, excluding low-complexity sequences based on a complexity threshold of 30, and retaining sequences that are at least 30 bases long. Then, bowtie2 (v2.4.5) (25) was used to align the high-quality sequence with the human reference genome build hg38 to remove the sequence of the human genome. The average removal rates of reads in fecal samples and saliva samples were 0.7% and 76.8%, respectively. Species annotation was performed using Kraken 2 (26), and the output results were subsequently corrected and quantified using Bracken (27). Gene functional annotation was performed using HUMAnN3 (28). We annotated antibiotic resistance genes using the ARGs_OAP pipeline (v3.2.4) (29).

### Statistical analysis

We performed all statistical analyses in R (V3.5.3; http://www.r-project.org/) and created data figures using the ggplot2 R package. Shannon index was measured using the vegan R package to quantify α-diversity. Beta diversity based on Bray-Curtis distance coupled with non-metric multidimensional scaling (NMDS) was calculated to indicate compositional differences and was tested by permutational multivariate analysis of variance (PERMANOVA) with the vegan R package. Phyla other than the nine phyla with the highest relative abundance were categorized as “Others.” To focus on the most prevalent and potentially impactful members of the microbiome, we selected the top 50 genera with the highest relative abundance to perform Kruskal-Wallis tests among groups using the stats R package, and genera with *P* < 0.05 were retained and visualized using the corrplot R package. It is important to note that both the gut and the oral microbiomes were not affected by sex, age, and BMI (Fig. S1), so subsequent analyses would not consider these factors. Furthermore, given that all participants consistently used the same immunosuppressants from R01 to R05, with dosages remaining basically consistent without significant fluctuations, which indicated that the use of immunosuppressants was a relatively constant factor and was unlikely to be the main driving force behind the changes in gut and oral microbiota. Also, although antibiotic use significantly affected the oral microbiome, the effect of kidney transplantation was much greater than that of antibiotic use for both the oral and gut microbiomes tested by the Adonis analysis. Therefore, it could be assumed that the microbial changes in this study were mainly caused by the transplantation itself (Fig. S2). The differences between HC and R00 were tested by Kruskal-Wallis tests. To avoid the bias caused by a small sample size in R05, the changes in microbial attributes after transplantation were fitted by nonlinear least squares, which indicated that we focused on the trends of changes after kidney transplantation rather than whether significant changes occurred at different stages. Microbial source tracking software Feast was used to predict the potential contributions to gut microbiomes from oral.

The SparCC algorithm was employed to construct co-occurrence networks by the SpiecEasi R package. We screened for species with more than 70% occurrence and the mean relative abundance greater than 0.001% to construct the networks. Then we applied filtering criteria to the SparCC results, retaining only correlations with an *R* > 0.7 and a false discovery rate <0.05. Network graphs were generated using the “igraph” package and were visualized using the Gephi platform. We extracted the subnetworks of the individual samples from the SparCC networks to calculate network properties, including modularity, degree, linkage density (degree/nodes), and centrality (eigenvector). This approach has been widely used when there are too few samples of a certain group to construct the network. We also used the membership function to count the module belongings of all nodes. Modules with more than 10% nodes were assigned numbers. In our microbial networks, nodes stand for individual microbial species, the basic units contributing to the community structure. Edges represent interactions between nodes, including positive or negative correlations that imply cooperative or competitive relationships. Modules are node clusters with denser internal connections. They may consist of microbes with similar ecological functions or responses to environmental changes. Furthermore, KOs with mean relative abundance greater than 0.001% were selected for correlation analysis with modules, and those with *P* < 0.05 and *R* > 0.5 were identified as related functions. These correlation networks were visualized using the Cytoscape platform. Moreover, a random forest model was used to predict estimated glomerular filtration rate (eGFR) and assess the relative importance of microbiome on eGFR to better understand the associations between microbial networks and kidney function. We used the CKD-EPI equation to calculate eGFR. Notably, due to the small sample size, we used 10-fold cross-validation and chose the best random forest model using the randomForest and caret R packages, then calculated the importance of each variable and the significance of the model using the rfPermute and A3 R packages.

## RESULTS

### Participant characteristics

Through the analysis of blood biochemistry indicators (Fig. 1b), we found that several kidney function-related indicators showed obvious changing trends before and after kidney transplantation. The results revealed that albumin (ALB), cholinesterase (CHE), indirect bilirubin (IBIL), K^+^, total protein (TP), and uric acid (UA) decreased first and then increased from R00 to R04, while Na^+^ showed an opposite trend (*P* < 0.05). Furthermore, blood urea nitrogen (BUN) and creatinine (CR) decreased, but Cl^−^ and gamma-glutamyl transferase (GGT) increased significantly after transplantation (*P* < 0.05). However, no significant variations were observed in alkaline phosphatase (ALP), aspartate aminotransferase (AST), direct bilirubin (DBIL), total bile acid (TBA), and total bilirubin (TBIL). Notably, CR, BUN, ALB, CHE, and TP approached or returned to normal levels, suggesting that kidney transplantation improved the kidney function and certain biochemical indicators of the patients.

### Alterations in taxonomic and functional diversity and composition of gut and oral microbiome

**Fig 2:**
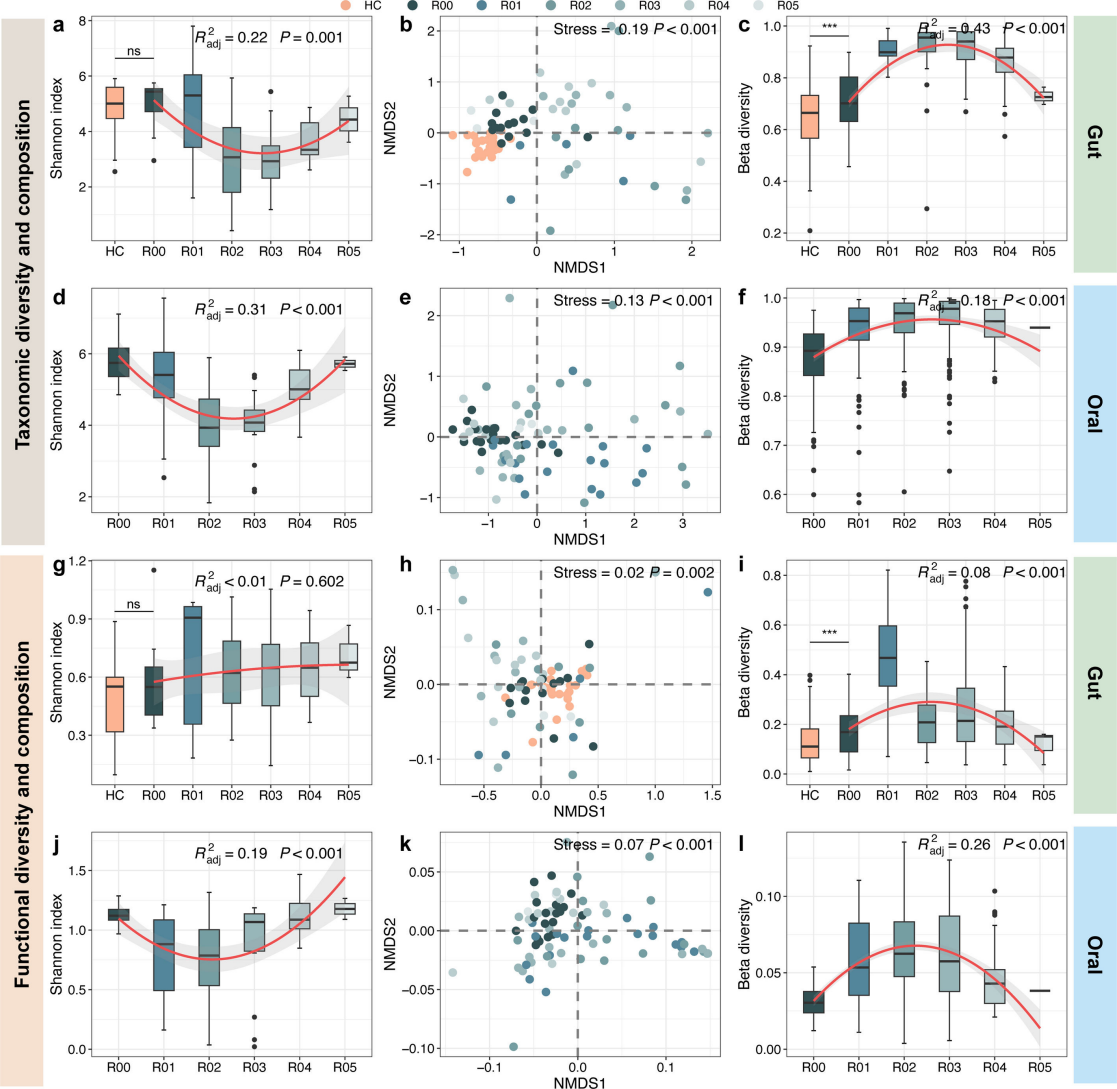
Changes in taxonomic and functional diversity and composition of gut and oral microbiome. The variations in taxonomic and functional alpha diversity (Shannon index) of gut (**a and d**) and oral microbiome (**g and j**), respectively. The variations in taxonomic and functional composition of gut (**b and e**) and oral microbiome (**h and k**), respectively. The variations in taxonomic and functional beta diversity of gut (**c and f**) and oral microbiome (**i and l**), respectively. These changes from R00 to R05 are fitted by nonlinear least squares. Asterisks indicate statistical significance between HC and R00, which is determined by Kruskal-Wallis tests (***P < 0.001, **P < 0.01, and *P < 0.05).

**Fig 3:**
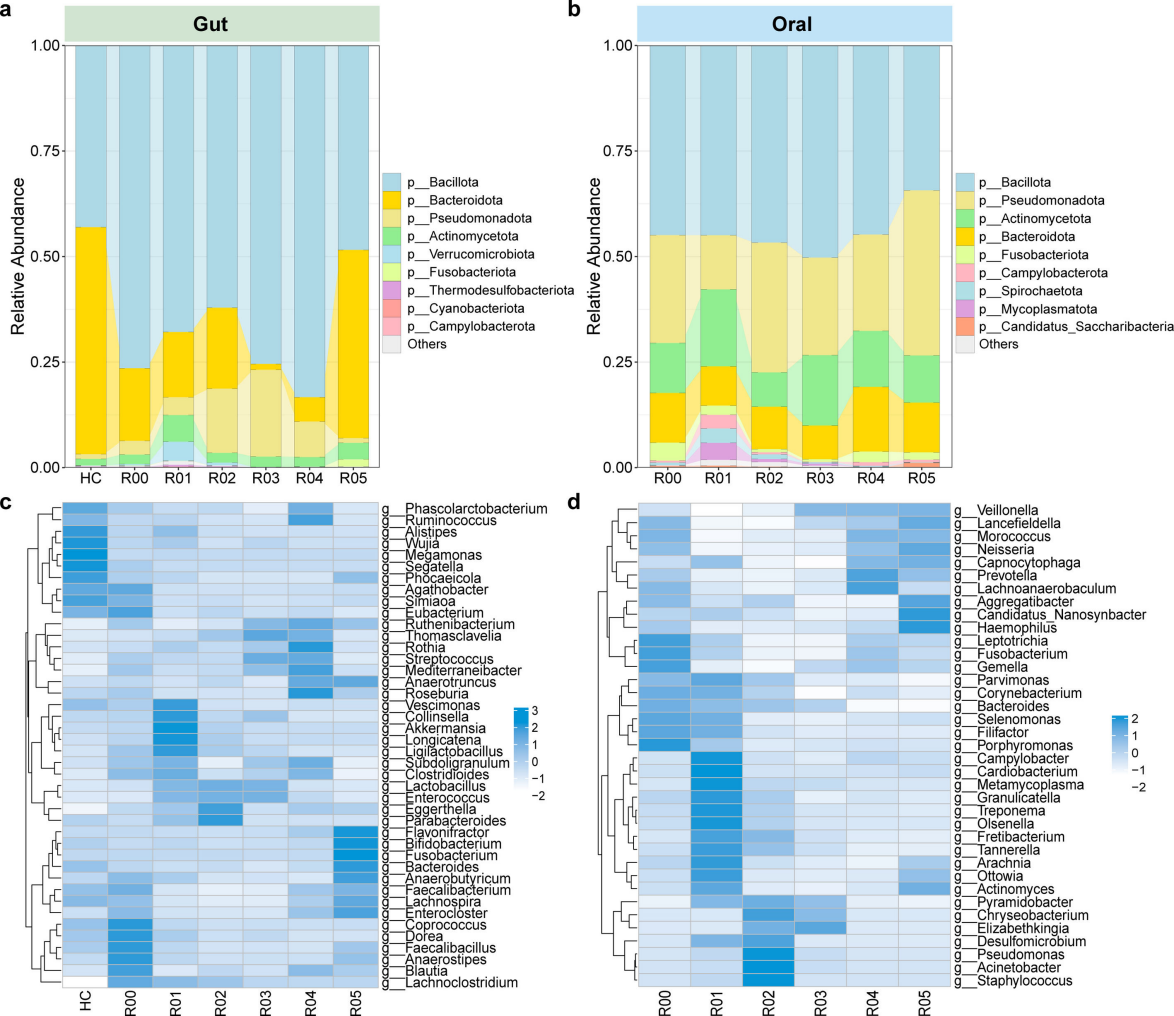
Changes in taxonomic composition of gut and oral microbiome at the phylum and genus levels. The variations in relative abundance of phyla in the gut (**a**) and oral microbiome (**b**), respectively. The variations in relative abundance of the genus in the gut (**c**) and oral microbiome (**d**), respectively.

As seen in Fig. 2a, taxonomic alpha diversity (Shannon index) of gut microbiome in HC did not significantly change compared to R00. However, NMDS showed that microbial communities formed different clusters between them (Fig. 2b), with higher beta diversity in patients with kidney failure (*P* < 0.05) (Fig. 2c). Moreover, as time after kidney transplantation progressed, both gut and oral communities exhibited obvious trends of first decreasing and then increasing in alpha diversity (Fig. 2a and d), as well as significant compositional alterations (Fig. 2b and e). However, their beta diversity (Bray-Curtis) displayed trends opposite to alpha diversity, that is, it increased first and then decreased (*P* < 0.05) (Fig. 2c and f). Furthermore, we found that the gut microbiome was dominated by Bacillota and Bacteroidota, which showed higher and lower relative abundances in R00 than HC, respectively. In contrast to Bacteroidota, a decrease followed by an increase in the abundance of Pseudomonadota was observed after transplantation (Fig. 3a). Oral microbiome was mainly composed of Bacillota, Pseudomonadota, Actinomycetota, and Bacteroidota, with the abundance of Bacillota increasing and then decreasing (Fig. 3b). At the genus level, the relative abundances of *Phascolarctobacterium*, *Ruminococcus*, *Alistipes*, *Wujia*, *Megamonas*, *Segatella*, and *Phocaeicola* were obviously higher in HC than R00, while those of *Dorea*, *Faecalibacillus*, *Anaerostipes*, *Blautia*, and *Lachnoclostridium* were in contrast and showed substantial decreases when the time point reached R05 (Fig. 3c). For the oral microbiome, we found that *Parvimonas*, *Bacteroides*, *Selenomonas*, *Filifactor*, and *Porphyromonas* displayed obvious decreases in their abundances as time progressed after transplantation (Fig. 3d). Interestingly, FEAST estimated percentages contributing to the gut microbiota by the microbiota from oral steadily decreased from 5.88% at R01 to 1.13% at R05 (Fig. S3).

**Fig 4:**
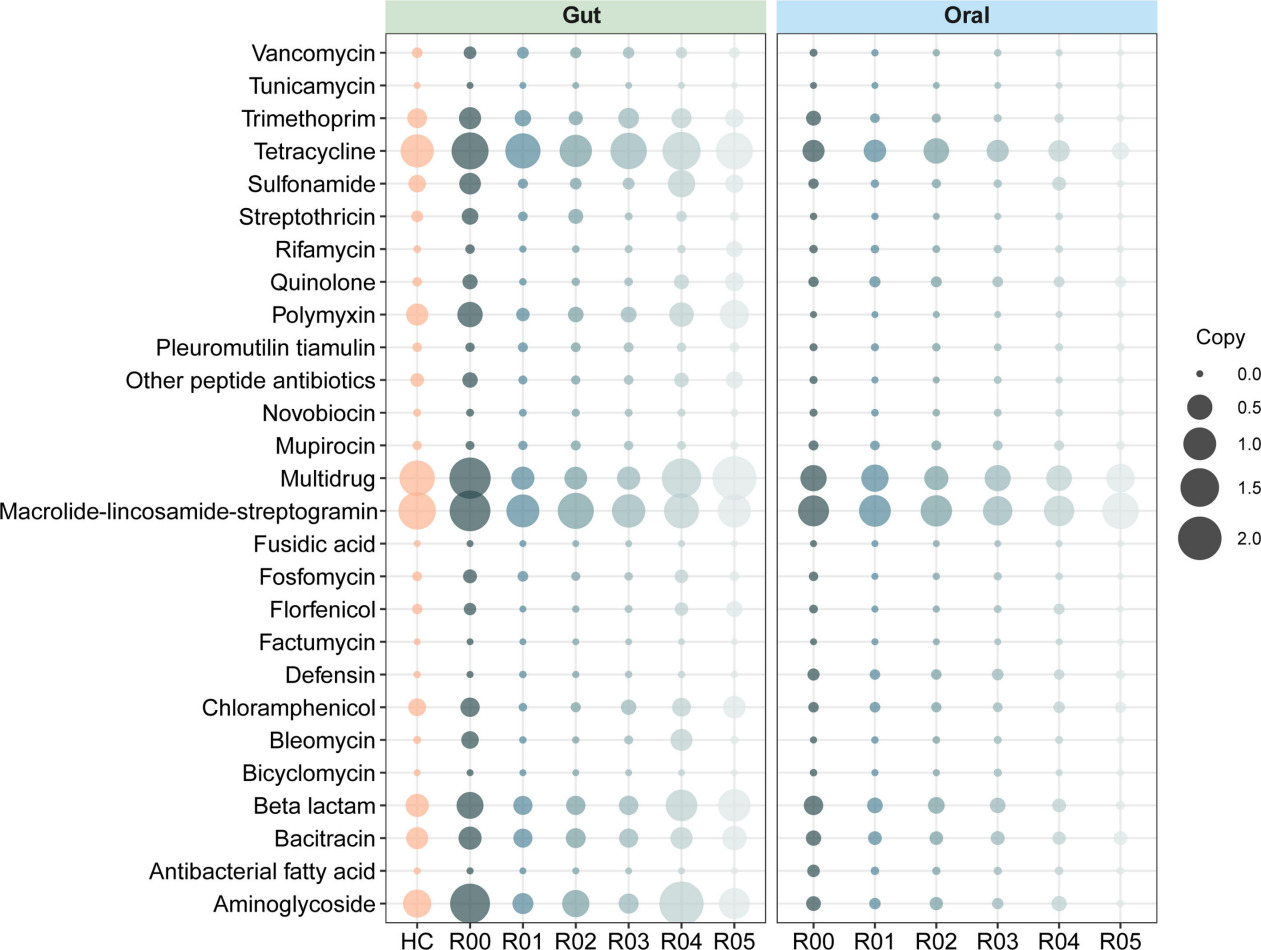
Changes in copies of ARGs in gut and oral microbiome. The variations in copies of ARGs in gut and oral microbiome, respectively.

We also examined the functional profiles of gut and oral microbiomes. The results suggested that functional alpha diversity did not vary significantly (Fig. 2g), but beta diversity was obviously higher in R00 than HC (*P* < 0.05) (Fig. 2h and i). From R00 to R05, functional beta diversity of both gut and oral microbiome exhibited initial increases followed by noteworthy declines (*P* < 0.05) (Fig. 2h, i, k and l). By contrast, alpha diversity of oral microbial functions showed an opposing trend (*P* < 0.05) (Fig. 2g), while no notable variations were observed in gut (Fig. 2j). Since patients were given antibiotics after transplantation, we focused on the changes in antibiotic resistance genes (ARGs). The results revealed that almost all ARGs in the gut microbiome had more copies in R00 than HC and exhibited initial decreases and subsequent increases from R00 to R05. This trend was particularly pronounced for the ARGs for tetracycline, polymyxin, multidrug, beta lactam, and aminoglycoside, which held the higher copies among all ARGs (Fig. 4). Although the copies of ARGs in the oral microbiome were relatively stable, we found that those for tetracycline beta-lactam, antibacterial fatty acid, and aminoglycoside displayed clear reductions after transplantation (Fig. 4). These results further supported the significant impact of kidney failure and transplantation on functions of gut and oral microbiome.

### Microbial co-occurrence networks and their related functions

**Fig 5:**
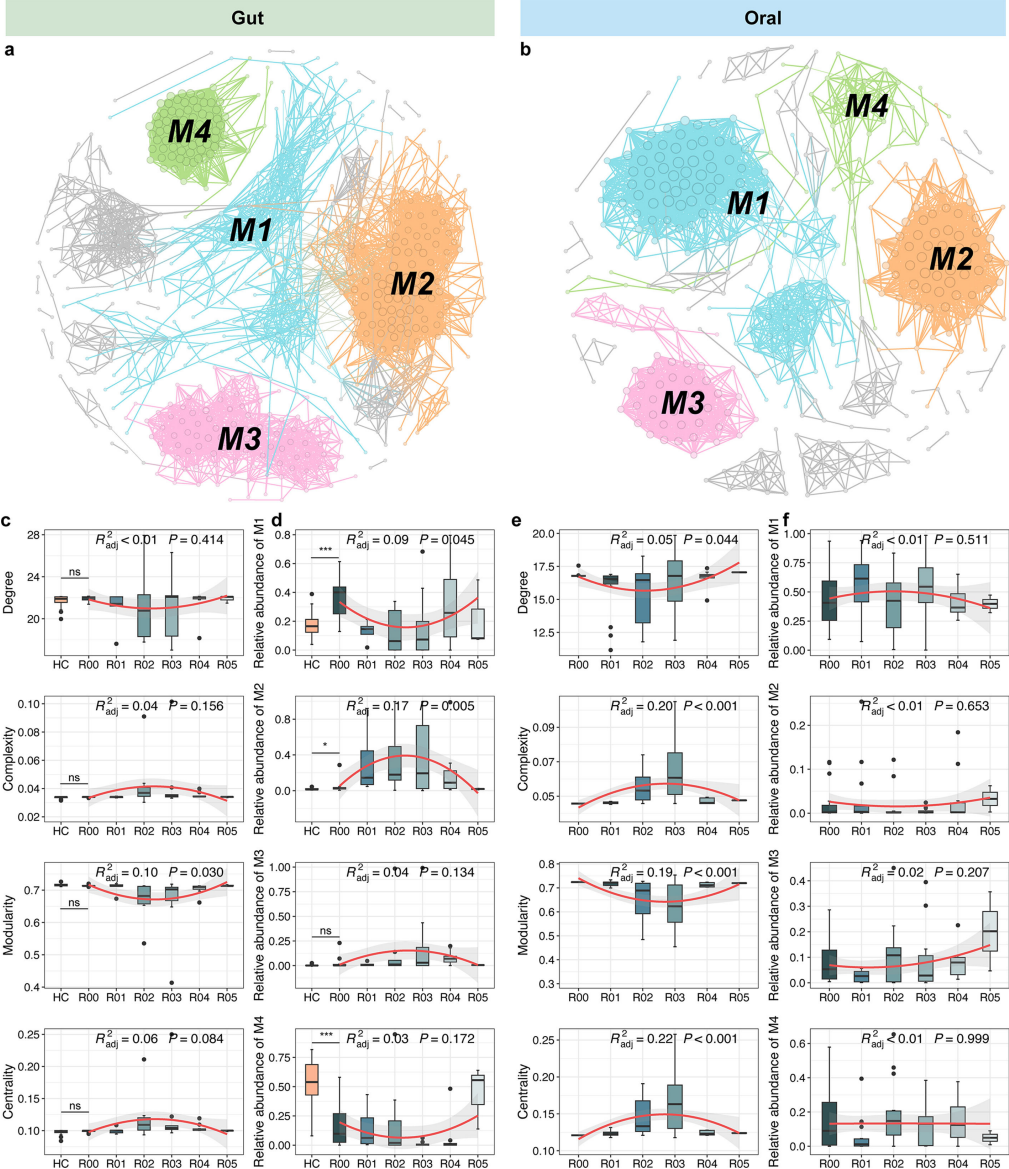
Changes in gut and oral microbial co-occurrence networks. The networks of gut (**a**) and oral (**b**) microbiome. The nodes were colored by module. The changes in network properties of gut (**c**) and oral (**e**) microbiome. The changes in relative abundances of M1–M4 in gut (**d**) and oral (**f**) microbiome. These changes from R00 to R05 are fitted by nonlinear least squares. Asterisks indicate statistical significance between HC and R00, which is determined by Kruskal-Wallis tests (***P < 0.001, **P < 0.01, and *P < 0.05).

Microbial co-occurrence networks were constructed based on the SparCC algorithm to evaluate the changes in possible ecological interactions among microbial members (Fig. 5a and b). For gut microbiome, we found that network properties, including degree, complexity (degree/node), and centrality (eigenvector), did not vary markedly between HC and R00, as well as from R00 to R05 (Fig. 5c). However, in oral microbial networks, degree and modularity exhibited declines followed by significant increases, while complexity and centrality displayed opposite trends as time progressed after transplantation (Fig. 5e), suggesting that the disappearance of certain microbial nodes in the short time after transplantation resulted in the loss of many interactions but also made the remaining members interact more closely. We also found that both the networks of gut and oral microbiome consisted mainly of four modules (M1–M4) (Fig. 5a and b). In the gut microbial network, the majority of members within M1 were affiliated with Bacillota and Thermodesulfobacteriota, while M2 was predominantly composed of species from Bacillota and Actinomycetota. By contrast, M3 and M4 were entirely composed of the species from Pseudomonadota and Bacteroidota, respectively (Fig. S4a). Parallelly, in the oral microbial network, species from Bacillota also dominated M1 and M2. Nevertheless, a substantial proportion of species in M1 originated from Spirochaetota, and those in M2 were mainly from Bacteroidota. The species in M3 primarily stemmed from Bacillota, Bacteroidota, and Fusobacteria, while all species in M4 belonged to Pseudomonadota (Fig. S4b). In terms of gut microbial network, R00 had higher relative abundances of M1 and M2 but lower abundance of M4 compared to HC. On the time gradient after transplantation, the abundance of M1 first decreased and then increased, while that of M2 was opposite (Fig. 5d). Differently, none of the four modules of the oral microbial network showed significant trends in changes (Fig. 5f). These results suggested the different responses of gut and oral microbial networks to kidney transplantation.

**Fig 6:**
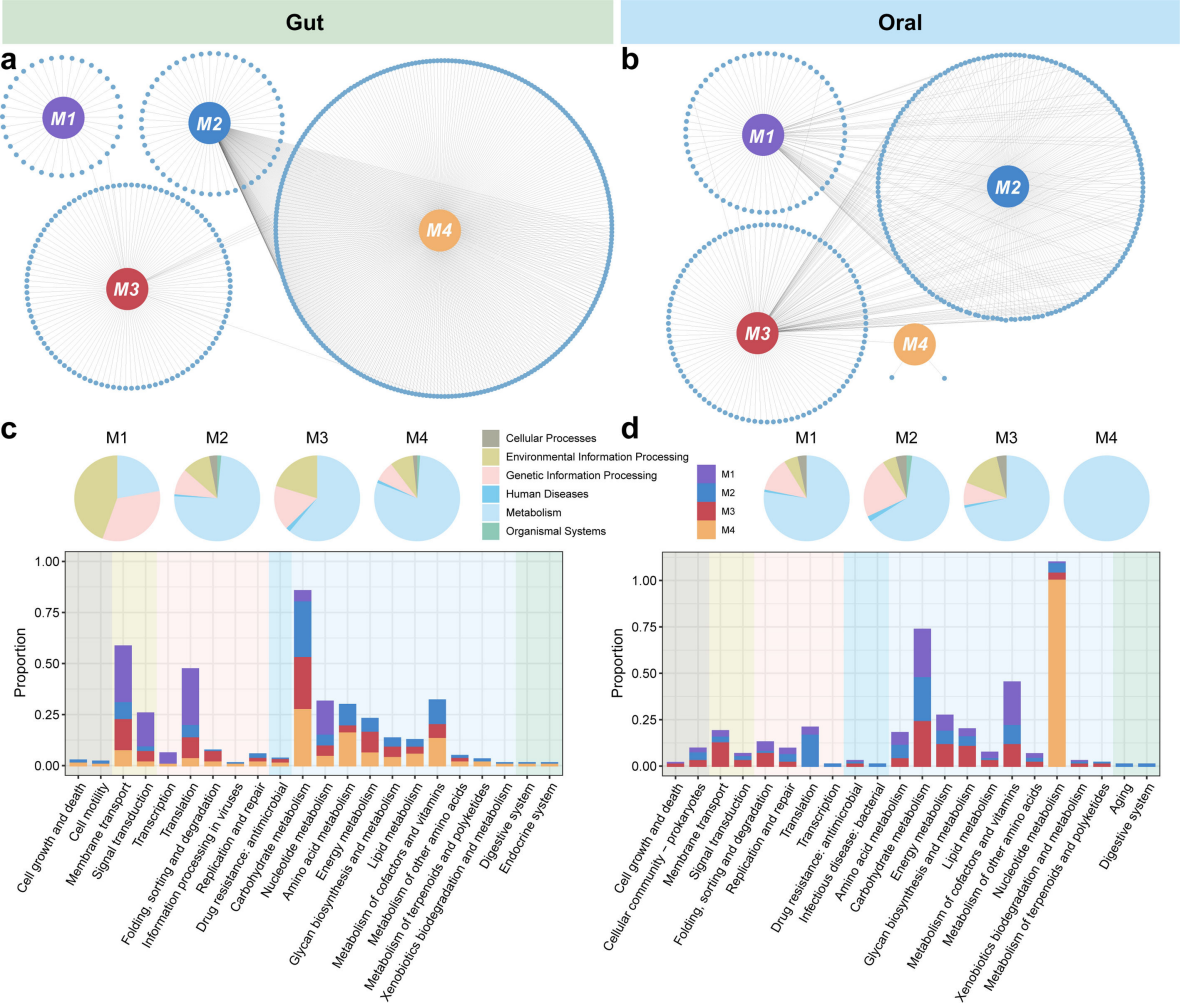
Functions related to microbial co-occurrence networks. The correlation networks between KEGG orthologs (KOs) and modules in gut (**a**) and oral (**b**) microbiome. The composition of modules related to functions in gut (**c**) and oral (**d**) microbiome.

We also explored the related functions of each module in gut and oral microbial networks (Fig. 6a and b). For gut microbial networks, most of the functions related to M1 belonged to environmental information processing and genetic information processing, mainly including membrane transport and translation. The functions performed by M2–M4 were dominated by metabolism, including carbohydrate metabolism, amino acid metabolism, energy metabolism, and metabolism of cofactors and vitamins (Fig. 6c). Regarding oral microbial networks, the functions associated with all four modules were primarily led by metabolism. Among them, M3 had a higher proportion of membrane transport, whereas M4 was only responsible for nucleotide metabolism (Fig. 6d). These results suggested that different taxa in the networks performed different functions in response to kidney transplantation.

### Associations between microbial networks and clinical indicators

**Fig 7:**
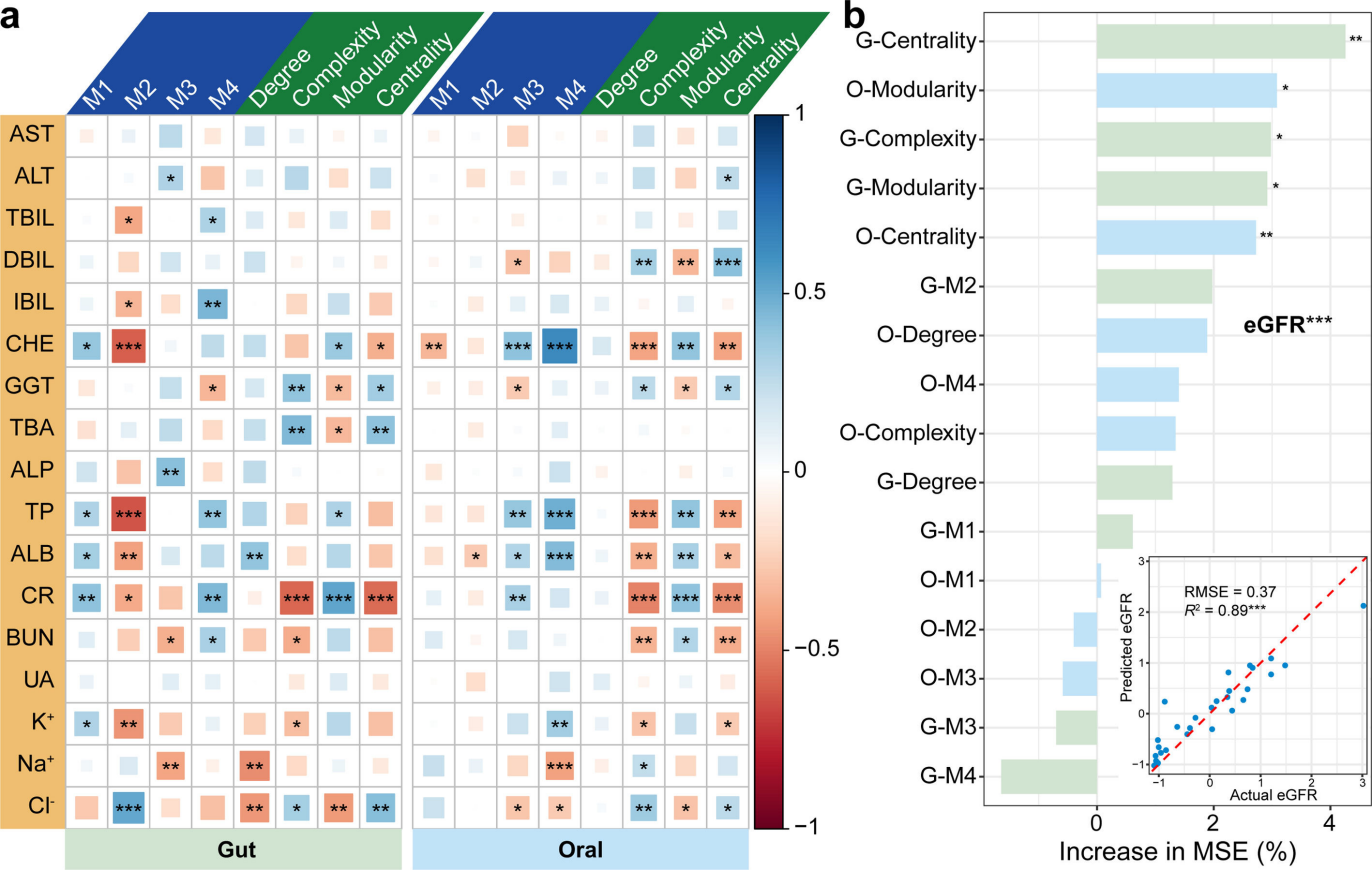
Associations between microbiome and clinical indicators. (**a**) The Spearman correlation of relative abundances of M1–M4 and network properties with clinical indicators in gut and oral microbiome. (**b**) Using a random forest model to predict eGFR and assess the relative importance of microbial networks on eGFR. RMSE, root mean squared error; MSE, mean squared error; Increase in MSE represents the importance of each variable on eGFR; Asterisks indicate statistical significance (***P < 0.001, **P < 0.01, and *P < 0.05).

We further examined the relationships between microbial networks and clinical indicators. The results showed that network complexity, modularity, and centrality in gut microbiome were all significantly associated with GGT, TBA, CR, and Cl^−^. Moreover, we found the relative abundance of M1 was significantly and positively linked to CHE, TP, ALB, CR, and K^+^, while the abundance of M2 exhibited pronounced negative associations with them (*P* < 0.05) (Fig. 7a), suggesting that M1 and M2 displayed completely opposite effects on the health of the organism after transplantation. For the oral microbiome, we obtained the same results that network complexity, modularity, and centrality were closely related to certain indicators (*P* < 0.05). Similarly, the abundance of M3 and M4 significantly influenced the variation of these indicators (*P* < 0.05) (Fig. 7a). Since the extent of kidney failure was determined by eGFR, we assessed the relative importance of gut and oral microbiomes on eGFR by random forest model. Based on the best model, we found that centrality, modularity, and complexity in gut microbial networks, as well as modularity and centrality in oral microbial networks, showed significant effects on eGFR (*P* < 0.05) (Fig. 7b). Meanwhile, the model could explain the high variances in eGFR (RMSE = 0.37, *R*^2^ = 0.89). These results suggested that the gut microbiome was more closely linked to kidney function than the oral microbiome after kidney transplantation, and microbial networks could capture the changes in eGFR well.

## DISCUSSION

In the current study, in patients with kidney failure, we identified the characteristics of the gut and oral microbiomes responding to kidney transplantation. We showed that for both gut and oral microbiome, the initial loss of species diversity after kidney transplantation led to a reduction in network nodes and interactions, but strengthened the connections among the remaining nodes, which started to recover after about 7–14 days. Also, modules in the network exhibited unique functions and different responses to transplantation. These network changes were strongly correlated with clinical indicators, suggesting that microbial networks play an important role in regulating kidney function and host health from dual dimensions after kidney transplantation.

As an important immune organ, the intestine regulates the host immune response and is the gathering place of immune cells. Metabolism-dependent and immune pathways co-mediate the gut-kidney axis (30). Consistent with a recent study (31), there were significant differences in gut microbial beta but not alpha diversity between healthy individuals and patients with kidney failure, suggesting that abnormal kidney function and individual differences may lead to significant changes in microbial community composition. For instance, the accumulation of metabolic wastes and toxins can alter the gut environment and decrease the abundance of beneficial bacteria that can produce short-chain fatty acids, such as *Phascolarctobacterium* (32), as demonstrated in our study. By contrast, *Lachnoclostridium* and *Anaerostipes* were enriched in the patients with kidney failure, which could be explained by the fact that these species have good anti-inflammatory effects in the face of inflammation (33, 34). After kidney transplantation, the observed first decrease and then gradual recovery in alpha diversity of both gut and oral microbiome can be attributed to the administration of prophylactic antibiotics during the perioperative period, such as latamoxef or imipenem (35, 36). Notably, although our study showed that antibiotic use affected oral and gut microbes, the extent of the effect was less than that of kidney transplantation itself, suggesting that microbial changes were primarily due to the transplantation (Fig. S2). Nevertheless, our results exhibited some changes in ARGs. The long-term pathological conditions of patients with kidney failure, along with prior exposure to certain antibiotic treatments, may have contributed to the adaptability of certain microbiota to antibiotics. During the initial period after transplantation, antibiotic use imposed selective pressure on microbes, resulting in a reduction or elimination of ARG-carrying bacteria. However, as microbial communities gradually adapted to the pressure of antibiotics and the gradual withdrawal of antibiotics, there was a subsequent rebound in the copies of ARGs. Moreover, the phylum-level findings in our study are in agreement with previous research (9, 37), showing that Bacillota dominates the gut microbiome in kidney transplant recipients. In addition, the increase in abundance of Pseudomonadota in the gut microbiome in the beginning after transplantation could be regarded as a marker of ecological microbiota imbalance or intestinal microbiota disorder (38). In terms of the oral microbiome, the enrichment of oral pathogens caused by kidney failure was significantly reduced after transplantation, such as *Parvimonas* (39), *Selenomonas* (40), and *Porphyromonas* (41), suggesting a potential improvement in oral microbial balance rather than definitive oral health improvement. Interestingly, we found that the contributions to the gut microbiota by oral microbiota decreased from R01 to R05, which reflected the “marker” hypothesis that the increased abundance of oral microbiota in the gut may be due to a decrease in the gut resident microbiota, rather than an increase in the abundance of oral microbiota themselves (42). These findings can be helpful to provide guidance for oral treatment after kidney transplantation.

A growing body of research is beginning to focus on the changes in microbial networks and their links with host health (21, 43, 44). This is because past studies mainly explored changes in the abundance of communities or species while ignoring the effects of interactions between microbial members (22). Various studies have suggested that gut microbial networks in healthy individuals show greater network connectivity and complexity, which can be attributed to the fact that a poorly developed network usually has lower functionality (22, 45). Our study demonstrated this, with dramatic fluctuations in network properties after kidney transplantation, both in gut and oral microbial networks. As a result of operations, antibiotic treatments, and other medical interventions (46), although the number of network nodes and degrees in the network decreased, the complexity and centrality showed an increase, suggesting that the remaining members became more connected to each other. This might indicate that the microbiome underwent a reorganization after surgery, forming a more compact and concentrated network structure. In our results, functional diversity did not change significantly after transplantation, possibly because these network changes maintained microbial functionality (47). Furthermore, a previous study has successfully used network features of gut microbiota to predict individual response to exercise and diet interventions (22). Also, our study demonstrated that network properties could well explain the changes in clinical indicators. For instance, our study showed that network complexity, modularity, and centrality in the gut microbiome were all significantly associated with GGT and TBA, which are biomarkers related to liver function. Liver dysfunction following kidney transplantation is a common complication, primarily attributed to drug-induced liver injury (48). Alterations in the topological properties of the gut microbial network reflect microbiota dysbiosis and recovery, which may influence the production of indoxyl sulfate and trimethylamine N-oxide (49). These metabolites require hepatic metabolism before renal excretion, and their long-term accumulation can exacerbate renal burden (50). In addition, kidney transplantation, as a major surgical procedure, involves general anesthesia and surgical trauma, both of which can impact liver function (51). Previous studies have confirmed that gut microbiota dysbiosis in patients with end-stage renal disease is significantly associated with abnormal liver enzymes, and reduced microbial diversity caused by the use of antibiotics and immunosuppressants post-transplantation is correlated with elevated markers of hepatotoxicity (51). These results reflect the interaction between the gut, liver, and kidney through various pathways. Our study highlights that, in addition to concentrating on the various species themselves, the topological properties of microbial networks hold significant importance as well.

The associations between microbial networks and host health are also reflected in the other dimension. In the field of environmental microbial ecology, many studies have shown that microbial networks can be divided into several important modules to perform specific functions (19). The same is true for the human ecosystem. Within the complex microbial communities, microbes form structural modules known as “guilds,” underpinning the unique functions that support host health (52). Based on this approach, past research has suggested that certain network groups are associated with diseases including obesity, type 2 diabetes, diabetic kidney disease, and diabetic neuropathy (21). Our study yielded similar results that the presence of specific modules in both gut and oral microbial networks was relevant to clinical indicators. For instance, CR exhibited significant positive and negative correlations with the abundances of M1 and M2 in gut microbial networks, respectively. That is, M2 might be a beneficial guild, while M1 tends to be a detrimental counterpart. Our study showed that a large part of the members in M1 were classified as Thermodesulfobacteriota, a phylum containing sulfate-reducing bacteria. Its metabolite H_2_S, in high concentration, can damage the integrity of the gut barrier, induce inflammation, and may aggravate the renal burden through the gut-renal axis (53). By contrast, the large number of species in Actinomycetota present in M2 may contain probiotics such as Bifidobacterium, which are essential for immune regulation and anti-infection (44). Furthermore, the major M1-related functions were membrane transport and translation, while carbohydrate metabolism was mainly performed by M2. This could be explained by the fact that acute rejection after operation may lead to an inflammatory response and cellular damage, and cells need increased membrane transport and protein synthesis to cope with the injury and repair damaged tissue (1, 54). Similarly, transplanted kidneys need to adapt to the new environment and blood supply in the short term, which may result in increased carbohydrate metabolism as cells require an increased energy supply to support cellular repair and regeneration (55), and this demand would decrease over time. In addition, recipients may receive nutritional supplementation to facilitate recovery, which may increase carbohydrate intake and metabolism (56). This pattern of coupling modules to function was also found in the oral microbial networks. As expected, due to the narrower functional range and variability of the oral microbiome (57), gut microbial networks in the gut played a more important role in predicting eGFR than those in the oral. Nevertheless, we cannot ignore the importance of the oral microbiome in synergizing with the gut microbiome in restoring kidney function and human health after kidney transplantation.

Our study provides novel insights into how gut and oral microbiomes respond to kidney transplantation in patients with kidney failure. However, there are certain limitations to our research. While our sample size is comparable to that of most studies, it remains relatively small, particularly for samples collected after half a year (R05). Although we minimized bias through methodological adjustments, it must be acknowledged that the interpretation of results in R05 requires caution, which is one of the limitations of our study. We also did not obtain oral and blood samples from corresponding healthy individuals, which might make our study incomplete. Furthermore, it should be noted that our study lacked technical negative controls (e.g., blank extractions), which could potentially affect our results due to contamination from reagents, laboratory procedures, or environmental sources. Therefore, incorporating negative controls in future studies will further strengthen the robustness of our conclusions. Although the host rate in the oral samples was high, it remained at a normal level. To further improve data accuracy, future research could employ more effective software tools (e.g., KneadData and BWA) and more precise genome references (e.g., human_T2T_index) to minimize contamination (58). Nevertheless, this study offers a new perspective on the investigation of human microbiota and their associations with host health. Utilizing the network properties of gut and oral microbiomes to predict eGFR or other clinical indicators may aid in assessing kidney function and recovery conditions following kidney transplantation. The approach is also expected to be applied to other disease cohorts, advancing the study of the microbiome and its clinical impact.

## Data Availability

The clean metagenome data generated in this study have been deposited in the CNSA of CNGBdb with accession code CNP0006573 (https://db.cngb.org/search/project/CNP0006573/). The STORMS checklist is available at https://doi.org/10.5281/zenodo.14232843.

## SUPPLEMENTAL MATERIAL

Supplemental figures msystems.00252-25-s0001.docx Fig. S1 to S4.

Supplemental tables msystems.00252-25-s0002.xlsx Tables S1 to S4.

ASM does not own the copyrights to Supplemental Material that may be linked to, or accessed through, an article. The authors have granted ASM a non-exclusive, world-wide license to publish the Supplemental Material files. Please contact the corresponding author directly for reuse.

## References

[1] García-Martínez Y, Borriello M, Capolongo G, Ingrosso D, Perna AF. 2023. The gut microbiota in kidney transplantation: a target for personalized therapy? Biology (Basel) 12:163. doi:10.3390/biology12020163 36829442 PMC9952448

[2] Bikbov B, Purcell CA, Levey AS, Smith M, Abdoli A, Abebe M, Adebayo OM, Afarideh M, Agarwal SK, Agudelo-Botero M, et al.. 2020. Global, regional, and national burden of chronic kidney disease, 1990–2017: a systematic analysis for the global burden of disease study 2017. The Lancet 395:709–733. doi:10.1016/S0140-6736(20)30045-3 PMC7049905 32061315

[3] Hariharan S, Rogers N, Naesens M, Pestana JM, Ferreira GF, Requião-Moura LR, Foresto RD, Kim SJ, Sullivan K, Helanterä I, Goutaudier V, Loupy A, Kute VB, Cardillo M, Tanabe K, Åsberg A, Jensen T, Mahillo B, Jeong JC, Anantharaman V, Callaghan C, Ravanan R, Manas D, Israni AK, Mehta RB. 2024. Long-term kidney transplant survival across the globe. Transplantation 108:e254–e263. doi:10.1097/TP.0000000000004977 38499511

[4] Opazo MC, Ortega-Rocha EM, Coronado-Arrázola I, Bonifaz LC, Boudin H, Neunlist M, Bueno SM, Kalergis AM, Riedel CA. 2018. Intestinal microbiota influences non-intestinal related autoimmune diseases. Front Microbiol 9:432. doi:10.3389/fmicb.2018.00432 29593681 PMC5857604

[5] Gabarre P, Loens C, Tamzali Y, Barrou B, Jaisser F, Tourret J. 2022. Immunosuppressive therapy after solid organ transplantation and the gut microbiota: bidirectional interactions with clinical consequences. Am J Transplant 22:1014–1030. doi:10.1111/ajt.16836 34510717

[6] Swarte JC, Zhang S, Nieuwenhuis LM, Gacesa R, Knobbe TJ, TransplantLines Investigators, De Meijer VE, Damman K, Verschuuren EAM, Gan TC, Fu J, Zhernakova A, Harmsen HJM, Blokzijl H, Bakker SJL, Björk JR, Weersma RK, TransplantLines investigators. 2024. Multiple indicators of gut dysbiosis predict all-cause and cause-specific mortality in solid organ transplant recipients. Gut 73:1650–1661. doi:10.1136/gutjnl-2023-331441 38955400

[7] Wu H, Singer J, Kwan TK, Loh YW, Wang C, Tan J, Li YJ, Lai SWC, Macia L, Alexander SI, Chadban SJ. 2020. Gut microbial metabolites induce donor-specific tolerance of kidney allografts through induction of T regulatory cells by short-chain fatty acids. J Am Soc Nephrol 31:1445–1461. doi:10.1681/ASN.2019080852 32482686 PMC7350991

[8] Guirong YE, Minjie Z, Lixin YU, Junsheng YE, Lin Y, Lisha S. 2018. Gut microbiota in renal transplant recipients, patients with chronic kidney disease and healthy subjects. Nan Fang Yi Ke Da Xue Xue Bao 38:1401–1408. doi:10.12122/j.issn.1673-4254.2018.12.01 30613005 PMC6744200

[9] Lee JR, Muthukumar T, Dadhania D, Toussaint NC, Ling L, Pamer E, Suthanthiran M. 2014. Gut microbial community structure and complications after kidney transplantation: a pilot study. Transplantation 98:697–705. doi:10.1097/TP.0000000000000370 25289916 PMC4189837

[10] Wu YH, Pan YP. 2023. Research progress in oral diseases and oral microbiota of organ transplant patients. Sichuan Da Xue Xue Bao Yi Xue Ban 54:61–65. doi:10.12182/20230160210 36647644 PMC10409052

[11] Lasisi TJ, Raji YR, Salako BL. 2016. Salivary creatinine and urea analysis in patients with chronic kidney disease: a case control study. BMC Nephrol 17:10. doi:10.1186/s12882-016-0222-x 26775026 PMC4715295

[12] Campbell PM, Humphreys GJ, Summers AM, Konkel JE, Knight CG, Augustine T, McBain AJ. 2020. Does the microbiome affect the outcome of renal transplantation? Front Cell Infect Microbiol 10:558644. doi:10.3389/fcimb.2020.558644 33425774 PMC7785772

[13] Diaz PI, Hong B-Y, Frias-Lopez J, Dupuy AK, Angeloni M, Abusleme L, Terzi E, Ioannidou E, Strausbaugh LD, Dongari-Bagtzoglou A. 2013. Transplantation-associated long-term immunosuppression promotes oral colonization by potentially opportunistic pathogens without impacting other members of the salivary bacteriome. Clin Vaccine Immunol 20:920–930. doi:10.1128/CVI.00734-12 23616410 PMC3675961

[14] Lee S, Arefaine B, Begum N, Stamouli M, Witherden E, Mohamad M, Harzandi A, Zamalloa A, Cai H, Williams R, Curtis MA, Edwards LA, Chokshi S, Mardinoglu A, Proctor G, Moyes DL, McPhail MJ, Shawcross DL, Uhlen M, Shoaie S, Patel VC. 2025. Oral-gut microbiome interactions in advanced cirrhosis: characterisation of pathogenic enterotypes and salivatypes, virulence factors and antimicrobial resistance. J Hepatol 82:622–633. doi:10.1016/j.jhep.2024.09.046 39447963

[15] Molyneaux PL, Mallia P, Cox MJ, Footitt J, Willis-Owen SAG, Homola D, Trujillo-Torralbo M-B, Elkin S, Kon OM, Cookson WOC, Moffatt MF, Johnston SL. 2013. Outgrowth of the bacterial airway microbiome after rhinovirus exacerbation of chronic obstructive pulmonary disease. Am J Respir Crit Care Med 188:1224–1231. doi:10.1164/rccm.201302-0341OC 23992479 PMC3863728

[16] Luo M, Zhu J, Jia J, Zhang H, Zhao J. 2024. Progress on network modeling and analysis of gut microecology: a review. Appl Environ Microbiol 90:e0009224. doi:10.1128/aem.00092-24 38415584 PMC11207142

[17] Uchiyama K, Naito Y, Takagi T. 2019. Intestinal microbiome as a novel therapeutic target for local and systemic inflammation. Pharmacol Ther 199:164–172. doi:10.1016/j.pharmthera.2019.03.006 30877020

[18] Matchado MS, Lauber M, Reitmeier S, Kacprowski T, Baumbach J, Haller D, List M. 2021. Network analysis methods for studying microbial communities: a mini review. Comput Struct Biotechnol J 19:2687–2698. doi:10.1016/j.csbj.2021.05.001 34093985 PMC8131268

[19] Gu Y, Dong C, Chen S, Jin J, Yang P, Chen J, Wei P, Bahadur A. 2024. Effect of soil archaea on N_2_O emission in alpine permafrost. Res Cold Arid Reg 16:45–62. doi:10.1016/j.rcar.2024.04.002

[20] Zhou G, Fan K, Li G, Gao S, Chang D, Liang T, Li S, Liang H, Zhang J, Che Z, Cao W. 2023. Synergistic effects of diazotrophs and arbuscular mycorrhizal fungi on soil biological nitrogen fixation after three decades of fertilization. Imeta 2:e81. doi:10.1002/imt2.81 38868350 PMC10989903

[21] Wu G, Xu T, Zhao N, Lam YY, Ding X, Wei D, Fan J, Shi Y, Li X, Li M, Ji S, Wang X, Fu H, Zhang F, Shi Y, Zhang C, Peng Y, Zhao L. 2024. A core microbiome signature as an indicator of health. Cell 187:6550–6565. doi:10.1016/j.cell.2024.09.019 39378879

[22] Cheng R, Wang L, Le S, Yang Y, Zhao C, Zhang X, Yang X, Xu T, Xu L, Wiklund P, Ge J, Lu D, Zhang C, Chen L, Cheng S. 2022. A randomized controlled trial for response of microbiome network to exercise and diet intervention in patients with nonalcoholic fatty liver disease. Nat Commun 13:2555. doi:10.1038/s41467-022-29968-0 35538056 PMC9091228

[23] Rao J, Peng L, Liang X, Jiang H, Geng C, Zhao X, Liu X, Fan G, Chen F, Mu F. 2020. Performance of copy number variants detection based on whole-genome sequencing by DNBSEQ platforms. BMC Bioinformatics 21:518. doi:10.1186/s12859-020-03859-x 33176676 PMC7659224

[24] Chen S. 2023. Ultrafast one-pass FASTQ data preprocessing, quality control, and deduplication using fastp. Imeta 2:e107. doi:10.1002/imt2.107 38868435 PMC10989850

[25] Langmead B, Salzberg SL. 2012. Fast gapped-read alignment with Bowtie 2. Nat Methods 9:357–359. doi:10.1038/nmeth.1923 22388286 PMC3322381

[26] Wood DE, Lu J, Langmead B. 2019. Improved metagenomic analysis with Kraken 2. Genome Biol 20:257. doi:10.1186/s13059-019-1891-0 31779668 PMC6883579

[27] Lu J, Breitwieser FP, Thielen P, Salzberg SL. 2017. Bracken: estimating species abundance in metagenomics data. PeerJ Comput Sci 3:3. doi:10.7717/peerj-cs.104 PMC12016282 40271438

[28] Beghini F, McIver LJ, Blanco-Míguez A, Dubois L, Asnicar F, Maharjan S, Mailyan A, Manghi P, Scholz M, Thomas AM, Valles-Colomer M, Weingart G, Zhang Y, Zolfo M, Huttenhower C, Franzosa EA, Segata N. 2021. Integrating taxonomic, functional, and strain-level profiling of diverse microbial communities with bioBakery 3. Elife 10:e65088. doi:10.7554/eLife.65088 33944776 PMC8096432

[29] Yin X, Jiang X-T, Chai B, Li L, Yang Y, Cole JR, Tiedje JM, Zhang T. 2018. ARGs-OAP v2.0 with an expanded SARG database and hidden markov models for enhancement characterization and quantification of antibiotic resistance genes in environmental metagenomes. Bioinformatics 34:2263–2270. doi:10.1093/bioinformatics/bty053 29408954

[30] Yang T, Richards EM, Pepine CJ, Raizada MK. 2018. The gut microbiota and the brain-gut-kidney axis in hypertension and chronic kidney disease. Nat Rev Nephrol 14:442–456. doi:10.1038/s41581-018-0018-2 29760448 PMC6385605

[31] Yan P, Luo S, Guo L, Wang X, Ren X, Lv J, Chen Y, Lin X, Chen J, Wang R. 2023. Unraveling intestinal microbial shifts in ESRD and kidney transplantation: implications for disease-related dysbiosis. Microorganisms 11:11. doi:10.3390/microorganisms11112747 PMC10673061 38004758

[32] Zhou X, Zhang B, Zhao X, Zhang P, Guo J, Zhuang Y, Wang S. 2023. Coffee leaf tea extracts improve hyperuricemia nephropathy and its associated negative effect in gut microbiota and amino acid metabolism in rats. J Agric Food Chem 71:17775–17787. doi:10.1021/acs.jafc.3c02797 37936369

[33] Zhang Q, Wu Y, Wang J, Wu G, Long W, Xue Z, Wang L, Zhang X, Pang X, Zhao Y, Zhao L, Zhang C. 2016. Accelerated dysbiosis of gut microbiota during aggravation of DSS-induced colitis by a butyrate-producing bacterium. Sci Rep 6:27572. doi:10.1038/srep27572 27264309 PMC4893749

[34] Zhang W-Q, Quan K-Y, Feng C-J, Zhang T, He Q-W, Kwok L-Y, Chen Y-F. 2022. The Lactobacillus gasseri G098 strain mitigates symptoms of DSS-induced inflammatory bowel disease in mice. Nutrients 14:3745. doi:10.3390/nu14183745 36145120 PMC9505107

[35] Swarte JC, Douwes RM, Hu S, Vich Vila A, Eisenga MF, van Londen M, Gomes-Neto AW, Weersma RK, Harmsen HJM, Bakker SJL. 2020. Characteristics and dysbiosis of the gut microbiome in renal transplant recipients. J Clin Med 9:386. doi:10.3390/jcm9020386 32024079 PMC7074359

[36] Chen G, Zhang X, Chen F. 2023. A cross-sectional study on gut microbiota in patients with chronic kidney disease undergoing kidney transplant or hemodialysis. Am J Transl Res 15:1756–1765. 37056836 PMC10086930

[37] Fricke WF, Maddox C, Song Y, Bromberg JS. 2014. Human microbiota characterization in the course of renal transplantation. Am J Transplant 14:416–427. doi:10.1111/ajt.12588 24373208

[38] Shin NR, Whon TW, Bae JW. 2015. Proteobacteria: microbial signature of dysbiosis in gut microbiota. Trends Biotechnol 33:496–503. doi:10.1016/j.tibtech.2015.06.011 26210164

[39] Zhao L, Zhang X, Zhou Y, Fu K, Lau HC-H, Chun TW-Y, Cheung AH-K, Coker OO, Wei H, Wu WK-K, Wong SH, Sung JJ-Y, To KF, Yu J. 2022. Parvimonas micra promotes colorectal tumorigenesis and is associated with prognosis of colorectal cancer patients. Oncogene 41:4200–4210. doi:10.1038/s41388-022-02395-7 35882981 PMC9439953

[40] Freire M, Nelson KE, Edlund A. 2021. The oral host-microbial interactome: an ecological chronometer of health? Trends Microbiol 29:551–561. doi:10.1016/j.tim.2020.11.004 33279381

[41] Jia L, Jiang Y, Wu L, Fu J, Du J, Luo Z, Guo L, Xu J, Liu Y. 2024. Porphyromonas gingivalis aggravates colitis via a gut microbiota-linoleic acid metabolism-Th17/Treg cell balance axis. Nat Commun 15:1617. doi:10.1038/s41467-024-45473-y 38388542 PMC10883948

[42] Liao C, Rolling T, Djukovic A, Fei T, Mishra V, Liu H, Lindberg C, Dai L, Zhai B, Peled JU, van den Brink MRM, Hohl TM, Xavier JB. 2024. Oral bacteria relative abundance in faeces increases due to gut microbiota depletion and is linked with patient outcomes. Nat Microbiol 9:1555–1565. doi:10.1038/s41564-024-01680-3 38698178 PMC11152985

[43] Guo S, Zhang H, Chu Y, Jiang Q, Ma Y. 2022. A neural network-based framework to understand the type 2 diabetes-related alteration of the human gut microbiome. Imeta 1:e20. doi:10.1002/imt2.20 38868565 PMC10989819

[44] Huang L, Pan G, Feng Y, Fan Z, Ma K, Wang R, Wang G, Huang G, Huang S, Hou Y, Han M, Xie L, Ma Y. 2023. Microbial network signatures of early colonizers in infants with eczema. Imeta 2:e90. doi:10.1002/imt2.90 38868421 PMC10989766

[45] Wagg C, Schlaeppi K, Banerjee S, Kuramae EE, van der Heijden MGA. 2019. Fungal-bacterial diversity and microbiome complexity predict ecosystem functioning. Nat Commun 10:4841. doi:10.1038/s41467-019-12798-y 31649246 PMC6813331

[46] Layeghifard M, Hwang DM, Guttman DS. 2017. Disentangling interactions in the microbiome: a network perspective. Trends Microbiol 25:217–228. doi:10.1016/j.tim.2016.11.008 27916383 PMC7172547

[47] Gao C, Bezemer TM, de Vries FT, van Bodegom PM. 2024. Trade-offs in soil microbial functions and soil health in agroecosystems. Trends Ecol Evol 39:895–903. doi:10.1016/j.tree.2024.05.013 38910081

[48] Lam PW, Wachs ME, Somberg KA, Vincenti F, Lake JR, Ferrell LD. 1996. Fibrosing cholestatic hepatitis in renal transplant recipients. Transplantation 61:378–381. doi:10.1097/00007890-199602150-00008 8610344

[49] Anand S, Mande SS. 2022. Host-microbiome interactions: gut-liver axis and its connection with other organs. NPJ Biofilms Microbiomes 8:89. doi:10.1038/s41522-022-00352-6 36319663 PMC9626460

[50] Ramezani A, Raj DS. 2014. The gut microbiome, kidney disease, and targeted interventions. J Am Soc Nephrol 25:657–670. doi:10.1681/ASN.2013080905 24231662 PMC3968507

[51] Swarte JC, Li Y, Hu S, Björk JR, Gacesa R, Vich Vila A, Douwes RM, Collij V, Kurilshikov A, Post A, et al.. 2022. Gut microbiome dysbiosis is associated with increased mortality after solid organ transplantation. Sci Transl Med 14:eabn7566. doi:10.1126/scitranslmed.abn7566 36044594

[52] Wu G, Zhao N, Zhang C, Lam YY, Zhao L. 2021. Guild-based analysis for understanding gut microbiome in human health and diseases. Genome Med 13:22. doi:10.1186/s13073-021-00840-y 33563315 PMC7874449

[53] Kushkevych I, Dordević D, Vítězová M. 2021. Possible synergy effect of hydrogen sulfide and acetate produced by sulfate-reducing bacteria on inflammatory bowel disease development. J Adv Res 27:71–78. doi:10.1016/j.jare.2020.03.007 33318867 PMC7728581

[54] Galetin A, Brouwer KLR, Tweedie D, Yoshida K, Sjöstedt N, Aleksunes L, Chu X, Evers R, Hafey MJ, Lai Y, Matsson P, Riselli A, Shen H, Sparreboom A, Varma MVS, Yang J, Yang X, Yee SW, Zamek-Gliszczynski MJ, Zhang L, Giacomini KM. 2024. Membrane transporters in drug development and as determinants of precision medicine. Nat Rev Drug Discov 23:255–280. doi:10.1038/s41573-023-00877-1 38267543 PMC11464068

[55] Fleseriu M, Biller BMK, Findling JW, Molitch ME, Schteingart DE, Gross C, SEISMIC Study Investigators. 2012. Mifepristone, a glucocorticoid receptor antagonist, produces clinical and metabolic benefits in patients with Cushing’s syndrome. J Clin Endocrinol Metab 97:2039–2049. doi:10.1210/jc.2011-3350 22466348

[56] Steinman TI, Becker BN, Frost AE, Olthoff KM, Smart FW, Suki WN, Wilkinson AH, Clinical Practice Committee, American Society of Transplantation. 2001. Guidelines for the referral and management of patients eligible for solid organ transplantation. Transplantation 71:1189–1204. doi:10.1097/00007890-200105150-00001 11397947

[57] Baker JL, Mark Welch JL, Kauffman KM, McLean JS, He X. 2024. The oral microbiome: diversity, biogeography and human health. Nat Rev Microbiol 22:89–104. doi:10.1038/s41579-023-00963-6 37700024 PMC11084736

[58] Gao Y, Luo H, Lyu H, Yang H, Yousuf S, Huang S, Liu Y-X. 2025. Benchmarking short-read metagenomics tools for removing host contamination. Gigascience 14:giaf004. doi:10.1093/gigascience/giaf004 40036691 PMC11878760

