# Supplementary material for: Dual associations of gut and oral microbial networks with kidney transplantation
Source: mSystems. 2025 Jul 9;10(8):e00252-25. doi: 10.1128/msystems.00252-25 (PMC12363170; doi:10.1128/msystems.00252-25)
Supplement: Supplemental figures — Fig. S1 to S4. [file msystems.00252-25-s0001.docx]

**Dual associations of gut and oral microbial networks with kidney transplantation**

**Supplementary Information**


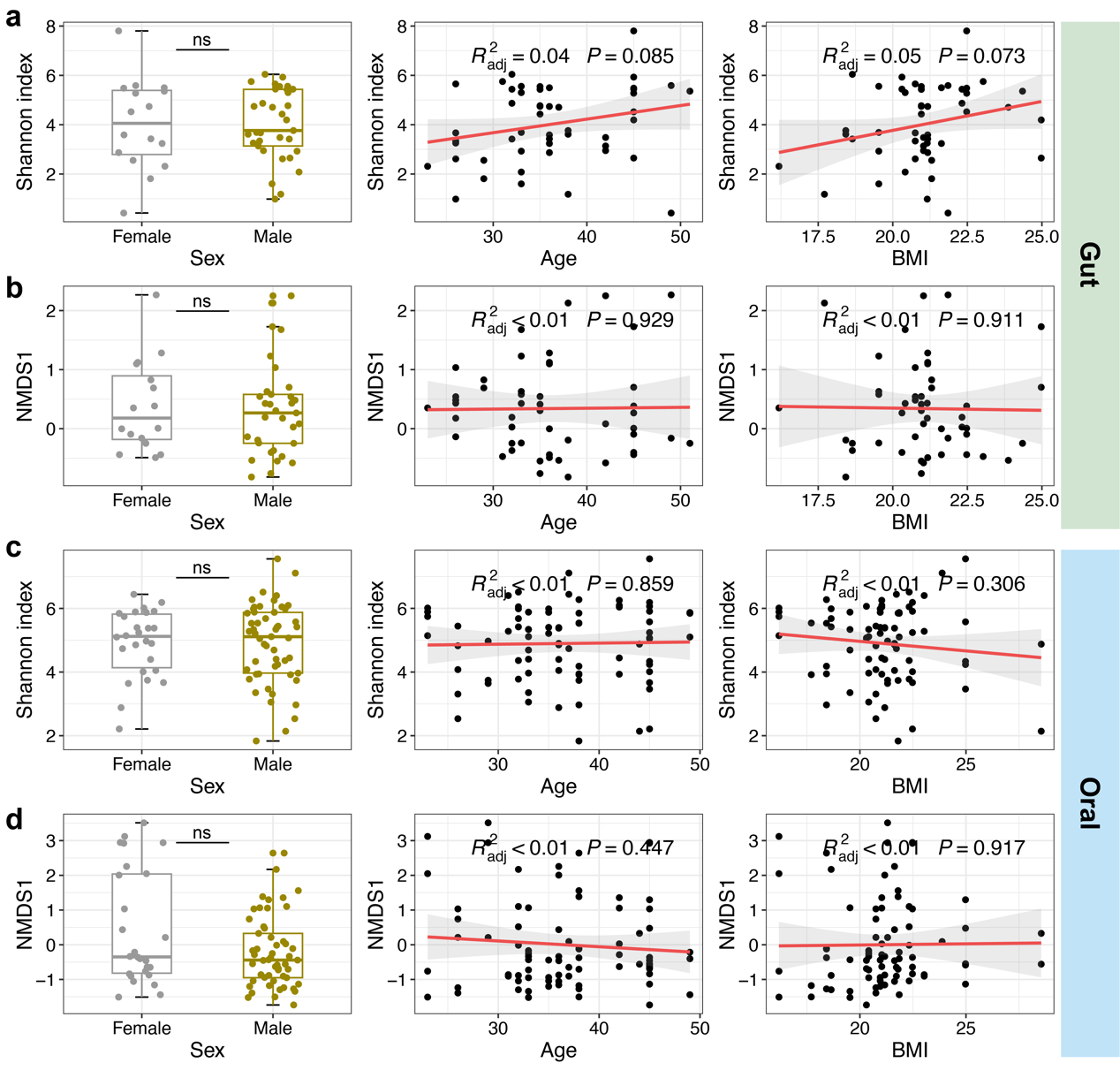


**Supplementary Fig. S1 Effects of sex, age, and BMI on gut and oral microbiome.** The associations of sex, age, and BMI with alpha diversity (Shannon index) and composition (NMDS1) of gut and oral microbiome.


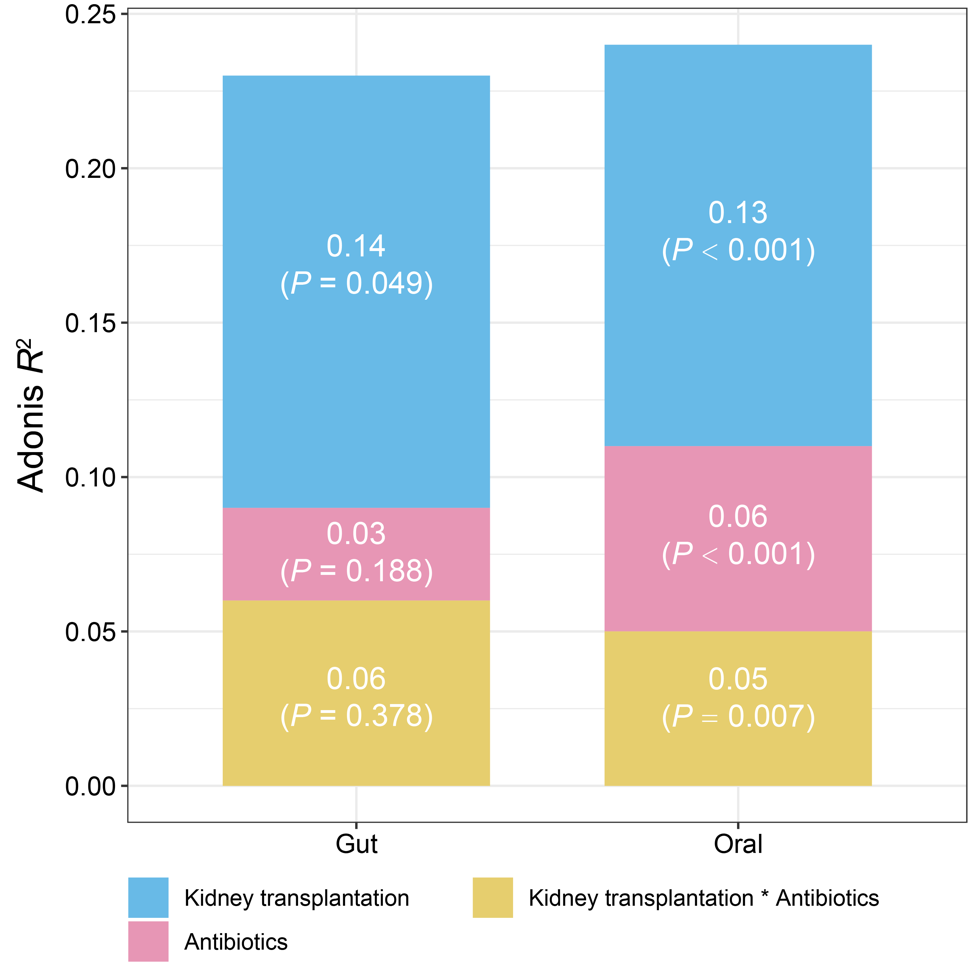


**Supplementary Fig. S2 Differences in the effects of kidney transplantation and antibiotics on gut and oral microbiome.** Bar plots illustrating the associations of kidney transplantation and antibiotics with gut and oral microbiome by their Adonis *R*^2^ values.


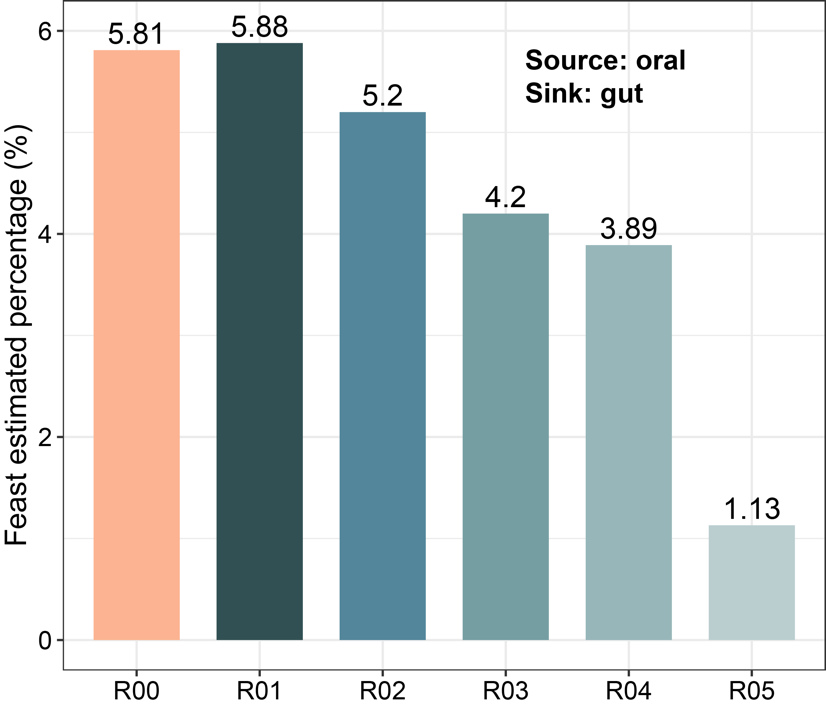


**Supplementary Fig. S3 Potential contribution of oral to gut microbiome.** FEAST estimated percentages contributing to the gut microbiome from oral sources.


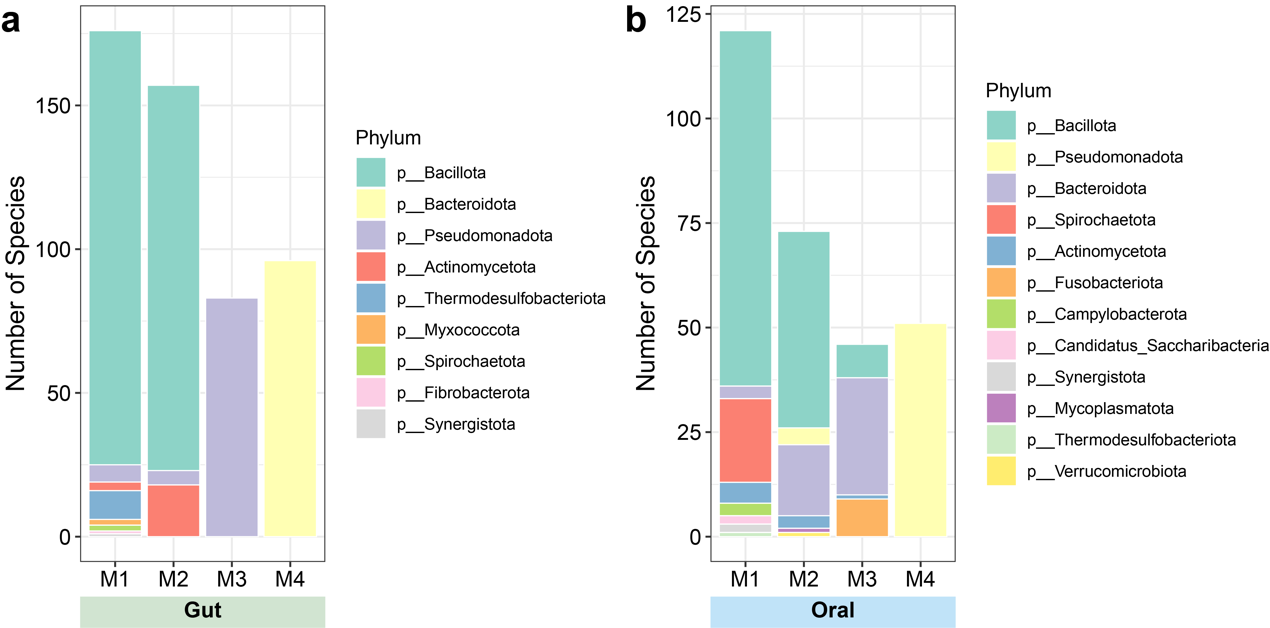


**Supplementary Fig. S4 Compositions of modules in gut and oral microbial networks.** Composition of M1 - 4 at phylum level in gut (a) and oral (b) microbial networks.
